# *In situ* phase formation during high-temperature synthesis in clad mechano­composites based on the Ti–Al system

**DOI:** 10.1107/S1600577522002004

**Published:** 2022-03-15

**Authors:** Marina Loginova, Alexey Sobachkin, Alexander Sitnikov, Vladimir Yakovlev, Andrey Myasnikov, Marat Sharafutdinov, Boris Tolochko, Tatiana Golovina

**Affiliations:** a Polzunov Altai State Technical University, Lenina Avenue, Barnaul, Altai Region 656038, Russian Federation; b Institute of Solid State Chemistry and Mechanochemistry of Siberian Branch Russian Academy of Sciences, Kutateladze, Novosibirsk 630128, Russian Federation

**Keywords:** precursors, self-propagating high-temperature synthesis, magnetron deposition, dynamic diffractometry

## Abstract

An *in situ* synchrotron experimental study of phase formation dynamics in clad mechano­composites of Ti–Al systems during high-temperature synthesis was performed. Cladding of the obtained mechano­composites was carried out with an SiO_2_ target, with a deposition time of 40 min. The high-temperature synthesis was performed using the thermal explosion method based on a microwave induction heater in the *in situ* mode on an experimental setup adapted to synchrotron radiation time-resolved diffractometry. The influence of the cladding on the macrokinetic parameters of synthesis *in situ* was investigated experimentally. It was found that for an ignition temperature *T*_ig_ = 650 ± 10°C, the maximum synthesis temperatures were in the range *T*_max_ = 1380–1465°C. The characteristic heating speed was 525 K min^−1^. The sequence and temperature–time interval of phase formation are determined. The formation of the TiAl, TiAl_3_ and Ti_3_Al compounds begins at *T* = 661°C. At *T*_max_ = 1465.6°C, the synthesis product is multiphase, the structures of the formed TiAl_3_ (content about 70%) and TiAl (content about 25%) have a nonequilibrium state. At the stage of system annealing with *T* = 1384.9°C, the reaction of the components occurs with the formation of almost monophase TiAl (content of more than 90%); Ti occupies the rest.

## Abstract 2

It is shown how cladding of mechano­composites with SiO_2_ film affects the course of high-temperature synthesis in Ti–Al systems.

## 1. Introduction

One of the important problems of modern powder metallurgy is obtaining composite powder materials with a complex of complementary properties for application in the field of gas-thermal methods of applying protective coatings, induction surfacing, obtaining structural materials, gas absorbers, *etc*.

One way of obtaining new materials using combustion processes is the method of self-propagating high-temperature synthesis (SHS), which has been used in many studies (Morsi, 2012 ▸; Li, 2012 ▸; Yi *et al.*, 1992 ▸; Li & Sekhar, 1993 ▸). The main advantages of this method are the simplicity of the equipment used, low energy consumption, and the short duration of the synthesis process. The process is based on conducting an exothermic chemical reaction of the initial reagents in the combustion form, where the target combustion products are solid chemical compounds (carbides, nitrides, borides, *etc*.) and materials based on them.

A classical SHS is synthesis in the dynamic thermal explosion mode, when the system is under ambient conditions and its temperature changes with time (Merzhanov *et al.*, 1977 ▸; Naiborodenko & Itin, 1976 ▸). Synthesis in the thermal explosion mode is usually carried out using a heating source, which only plays the role of the initiator of the reaction. However, synthesis in the thermal explosion mode can be carried out by microwave heating of the mixture (Shon *et al.*, 2007 ▸; Zhu *et al.*, 2010 ▸; Gedevanishvili *et al.*, 1999 ▸; Naplocha & Granat, 2009 ▸). In this case, it becomes possible to control the heating of the mixture by heating a crucible (usually a graphite one) with the mixture inside. Owing to the high heating efficiency, the mixture can be heated at high speed up to high temperatures, which is extremely important. It is also possible to cool the system quickly to room temperatures. Microwave heating makes it possible to trace the structural and phase composition of the synthesized materials at high ambient temperatures (possibly above adiabatic combustion temperatures) without cooling (forced thermal mode), which can maintain high temperatures (up to 1800°C) after the synthesis process. An effective way to obtain binary and multicomponent compounds in the form of powders is to combine the SHS method with mechanically activated pretreatment (MA SHS) (Bernard & Gaffet, 2001 ▸; Filimonov *et al.*, 2010 ▸; Rogachev & Mukasyan, 2015 ▸). First, using specialized mills, powdered reagents in the solid phase are mixed, forming a matrix structure, which makes it possible to form an ideal contact between the reagents and increases the reactivity of the mixture (Yadav *et al.*, 2012 ▸). The basis of the mechano­composites formed during the milling process is a metal matrix with a plastic component, within the volume of which are more brittle elements (Mukasyan *et al.*, 2011 ▸). Thus, a mechano­composite is a certain independent structural element within the volume of which heterogeneous synthesis reactions can occur. As shown by Manukyan *et al.* (2012 ▸), a mechano­composite can be called a ‘microreactor’, with characteristic external dimensions of 50–600 µm. Then, the process of high-temperature synthesis is realized based on the formed precursors.

It has been established that, in a number of binary systems, the combustion reaction is initiated in the solid phase (Shteinberg *et al.*, 2010 ▸; White *et al.*, 2009 ▸). This is influenced by mechano­activation pretreatment, which contributes to an increase in the specific surface area of the reactants, purification from oxides and impurities, and the formation of nonequilibrium structure defects.

In many solid-phase reactions, the formation of intermediate products is observed. These products then react with the initial components or with each other and give the final product of the reaction. Naturally, such solid-phase reactions are divided into several stages, with a mechanism and kinetics characteristic of each of them. The synthesis processes can be significantly influenced by the size and shape of the reacting particles, the degree of the particles mixture homogeneity, the composition of the gas phase, temperature, *etc.* (Filimonov *et al.*, 2013 ▸; Curfs *et al.*, 2007 ▸; Gras *et al.*, 2006 ▸).

Another factor that can affect the course of the reaction is surface modification of the prepared precursors – mechano­composites – by depositing thin films on them. It should be noted that the deposition of thin films on particles has become one of the more promising directions in the modification of powder materials (Xu *et al.*, 2007 ▸; Akamaru *et al.*, 2006 ▸; Shishkin *et al.*, 2018 ▸; Lv *et al.*, 2009 ▸; Baechle *et al.*, 2013 ▸).

The use of clad powders in the technologies of obtaining new composite materials and coatings can solve the problem of neutralizing the particles’ interaction with a gas environment during heating. One of the most promising methods of obtaining such powder materials is the method of magnetron sputtering, which makes it possible to create composite powder materials by depositing thin films on them (Ramaseshan *et al.*, 2001 ▸; Smirnov *et al.*, 2017 ▸; Schmid & Eisenmenger-Sittner, 2013 ▸; Braun, 2015 ▸).

The advantages of magnetron sputtering are high productivity, chemical composition accuracy of the deposited substance, uniformity of the coating, and the absence of thermal effects on the processed material (Jameel, 2015 ▸; Wasa *et al.*, 2004 ▸; Hovsepian *et al.*, 2007 ▸).

From the point of view of scientific novelty, the formation of mechano­composites clad with a protective shell is an urgent task for carrying out high-temperature synthesis. In this work, the Ti–Al system, which has wide application in the technologies of modern materials science, is investigated using model experiments (Sina & Iyengar, 2015 ▸; Chin & Biederman, 1992 ▸; Okamoto, 1993 ▸; Lee *et al.*, 2000 ▸).

Loginova *et al.* (2019*a* ▸) studied in detail the effect of preliminary mechanical activation of a Ti + Al powder mixture on the processes of high-temperature synthesis. In this regard, it is of particular interest to conduct model experiments to study the effect of Ti + Al mechano­composites cladding on phase formation and thermal synthesis processes.

An understanding of high-rate processes can be achieved through synchrotron radiation time-resolved diffractometry. The high speed of diffraction registration (which makes it possible to register high-rate processes), high synchrotron radiation intensity, and high spatial and time resolution of the detector provide high accuracy and make it possible to detect phase transitions in the processes of synthesis, deformation, melting, crystallization, *etc*.

Based on the above, the purpose of the present studies is to determine the effect of a SiO_2_ shell deposited on Ti + Al mechano­composites on the phase formation processes and thermal characteristics of high-temperature synthesis by conducting model experiments.

## 2. Experimental methodology

A mechanically activated (MA) and then clad Ti (64 wt%) + Al powder composition was used as the object of experimental study. The activation was carried out using an AGO-2 planetary ball mill with two cylinders with a volume of 160 cm^3^. The powder mass in each cylinder was 10 g. To prevent oxidation, the cylinders were evacuated and then filled with argon to a pressure of 0.3 MPa. The mass ratio of the initial powder mixture to the mass of the grinding bodies was 1:20, the centripetal acceleration was 40*g*, and the duration of mechanical activation was 7 min (Filimonov *et al.*, 2019 ▸, 2020 ▸). Cladding of the obtained mechano­composites was performed using an SiO_2_ target on a VSE-PVD-Power magnetron setup for spraying thin films in vacuum. In this setup, the mechanism of cuvette movement from a vibromechanism is implemented. Rotation speed is up to 1000 rev min^−1^. This solution makes it possible to achieve maximum mixing of powder particles during the magnetron sputtering process. The vacuum chamber of the setup has double water-cooled walls. To prevent excessive heating of the SiO_2_ target during ion bombardment, an air–water cooling mechanism of the holder is used.

The pumping time of the working chamber from atmospheric to working (1 × 10^−5^ Pa) is 30 min. The location of the magnetron is on the top of the vacuum chamber on the flanges. Pumping is carried out using an ISP-250-SV ‘Anest Iwata’ pre-vacuum pump, with pumping speed 250 l min^−1^, and a TMP-403LM ‘Shimadzu’ high-vacuum turbomolecular pump, with pumping speed 400 l s^−1^. Pressure is controlled by means of fore-vacuum (up to pressure 10^−4^ Torr) and high-vacuum (up to pressure 10^−9^ Torr) sensors connected directly to the chamber volume. The working gas is argon.

The coating deposition time, τ, was 40 min. A series of pre­liminary experiments was carried out in order to identify the dependence of the thickness of the deposited SiO_2_ film on the Ti + Al mechano­composite on the magnetron sputtering time at constant velocity values. At deposition times of 10 min, 20 min and 40 min, film thicknesses of 1.3 µm, 2.6 µm and 5.2 µm, respectively, were obtained. The thickness of the deposited coating was measured using a certified SQM-160 device designed to measure the thickness and the rate of film sputtering. The proven technology of an INFICON sensor based on a quartz crystal was used to measure the sputtering rate and layer thickness in the thin-films sputtering processes.

**Figure 1:**
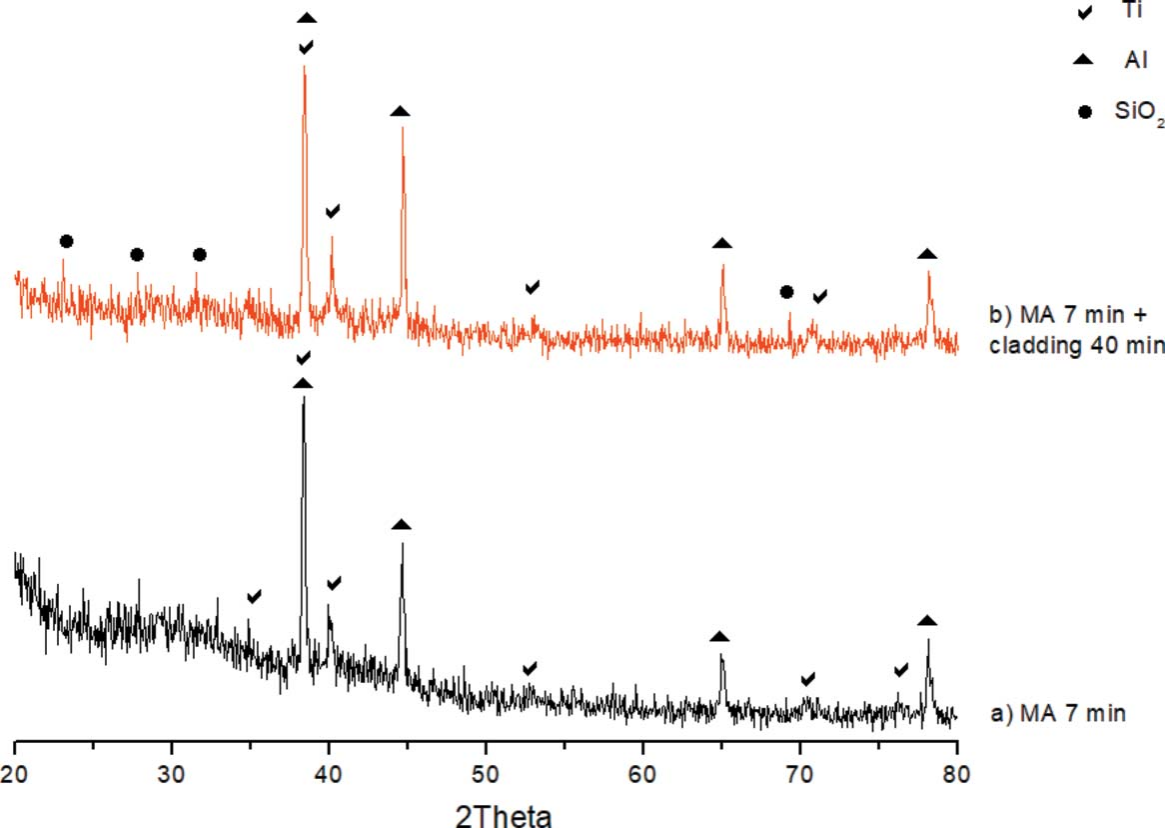
Diffractograms of the Ti (64 wt%.) + Al mechano­composite (*a*, bottom) and the mechano­composite after cladding with SiO_2_ target (*b*, top)

Fig. 1 ▸ shows diffractograms of a Ti (64 wt%) + Al mechano­composite and a mechano­composite of the same composition after cladding with SiO_2_ target. The additional peaks that appeared after cladding, corresponding to the interplanar distances *d* = 3.85 Å, 3.2 Å and 2.82 Å, are identified as silicon dioxide, SiO_2_. An SiO_2_ film of polycrystalline structure was formed with the orientation in the crystallographic directions (1 0 19), (2 1 3) and (2 1 4), respectively (card 14–260; Hill & Roy, 1958 ▸; PDWin 3.0 International Powder Diffractometry Database).

**Figure 2:**
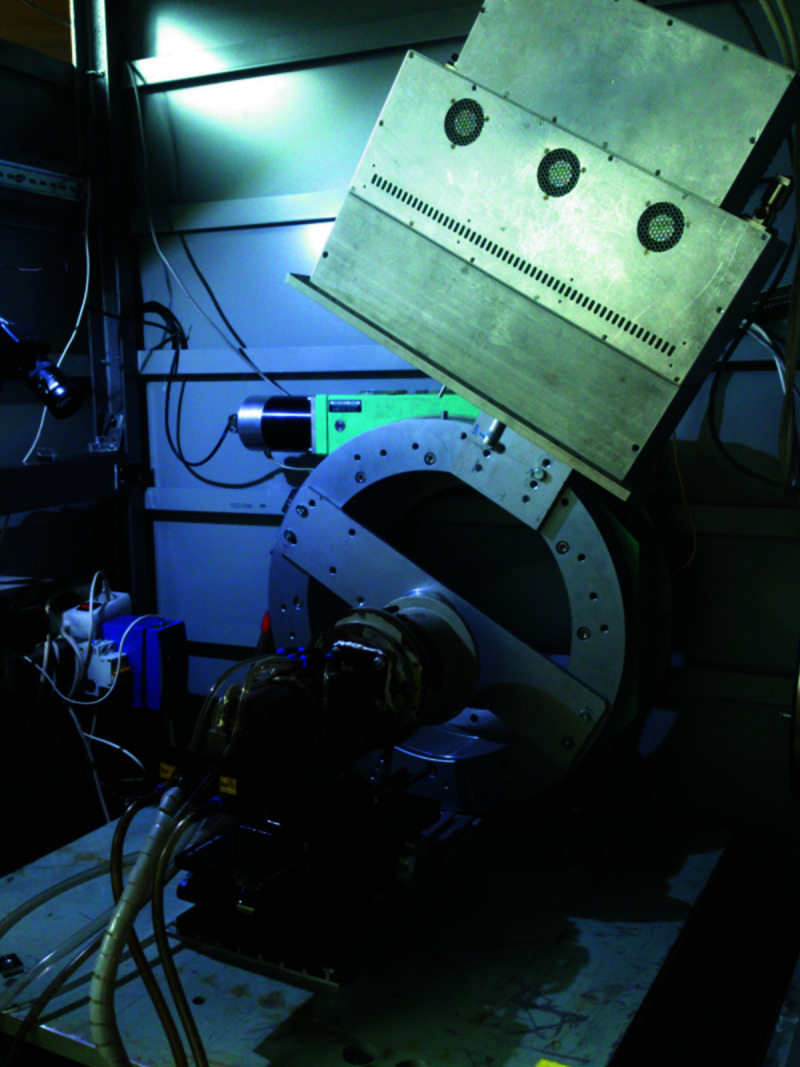
Working station 5b ‘diffraction movie’ at VEPP-3 with adapted experimental setup for study of phase formation dynamics during high-temperature synthesis in thermal explosion mode.

*In situ* high-temperature synthesis of the obtained clad mechano­composites in thermal explosion mode was performed using synchrotron radiation time-resolved diffractometry (TRXRD). Experiments were carried out at the VEPP-3 5b station at Budker Institute of Nuclear Physics of Siberian Russian Academy of Sciences, Novosibirsk, Russia (Evdokov *et al.*, 2009 ▸; Piminov *et al.*, 2016 ▸) (Fig. 2 ▸). TRXRD makes it possible to carry out sequential shooting of a series of diffractograms and to observe phase transformations in a substance in the processes of deformation, melting, crystallization, synthesis, *etc.* (Loginova *et al.*, 2019*b* ▸).

Time-resolved powder diffraction measurements were performed using an experimental setup based on the microwave inductor SVCh-6A with the possibility of rapidly heating the powder mixture. The maximum power of the inductor is 6 kW. The use of the microwave inductor makes it possible to conduct rapid heating of the mixture to high temperatures permanently, system exposure at high temperatures immediately after synthesis (high-temperature annealing), and rapid cooling of the mixture to room temperatures. This technology provides a controlled thermal effect on the powder mixture (Filimonov *et al.*, 2014*a* ▸).

**Figure 3:**
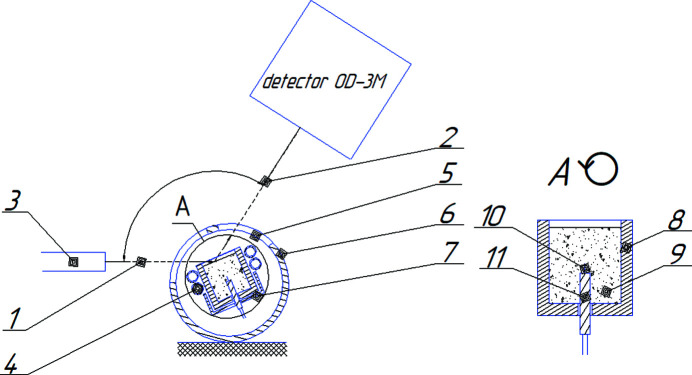
Scheme of the experimental setup: 1 – incident synchrotron radiation beam; 2 – reflected synchrotron radiation beam; 3 – emitter; 4 – inductor spiral; 5 – beryllium window; 6 – vacuum chamber; 7 – crucible holder; 8 – crucible; 9 – powder reagents; 10 – thermocouple; 11 – isolator.

The created experimental setup was adapted for use with synchrotron radiation. Fig. 3 ▸ shows a schematic of the VEPP-3 storage workstation 5b with experimental setup based on an induction heater.

A powder mixture consisting of the Ti + Al clad mechano­composites was filled into and compacted in the volume of a cylindrical graphite crucible. Then the crucible was placed in the inductor. The system was installed under a vacuum cap, under which air was pumped out to a pressure of 0.1 atm; then argon was injected to a pressure of 0.8 atm. Microwave heating of the crucible was carried out using an induction coil. To measure the heating temperature of powder mixtures over a wide temperature range, the most optimal method is the thermocouple method of temperature determination. Temperature control was carried out with the help of a tungsten–rhenium thermocouple, type VR-5/20, which was placed in the center of the powder mixture. To register the charge temperature in the reactor, a small-sized portable device for multi-channel temperature registration was used. Its basis is the analog-to-digital converter LA-2USB. The device allows signals from thermocouples to be received, converts them into measured temperature values, displays them on a computer monitor in real time, and saves the obtained data onto computer hard disk. A distinctive feature of the device is its ability to register the temperature by use of thermocouple without compensation junction. Temperature compensation was performed programmatically by entering the ambient temperature value into the corresponding program setup window. The device provides the possibility of using tabular calibration graphs for the thermocouple used, which show the dependence of the thermocouple voltage readings on temperature.

The process of high-temperature synthesis was initiated by increasing the inductor power by heating the mixture in fast variable electromagnetic fields with simultaneous temperature registration, which was displayed in real time on a computer screen.

Simultaneously with the start of synthesis initiation, the shooting process was started, and automatic registration of diffractograms was turned on. An OD-3M non-parallax one-coordinate detector with a 350 mm focal length was used (Aulchenko *et al.*, 2009 ▸). The detector has 3328 channels, registration angle ∼30°, maximum loading ∼10 MHz, minimum frame time ∼1 µs, and maximum number of frames of 64 (at maximum angular resolution). The instrumental error of the detector, equal to half of the division price of one channel, is 0.6%.

Since the detector has a fixed registration angle range of about 30°, the angular range was chosen taking into account the angular location of the main phases of titanium aluminides and was set to 34–66°. The diffraction patterns were recorded frame by frame. The one-frame time was set to 2 s.

Registration and subsequent processing of the diffractograms were carried out using the program *dqv2* developed at INP SB RAS (Novosibirsk), which made it possible to control the experiment, and to visualize, process and store the obtained data.

## 3. Results of the experiment

**Figure 4:**
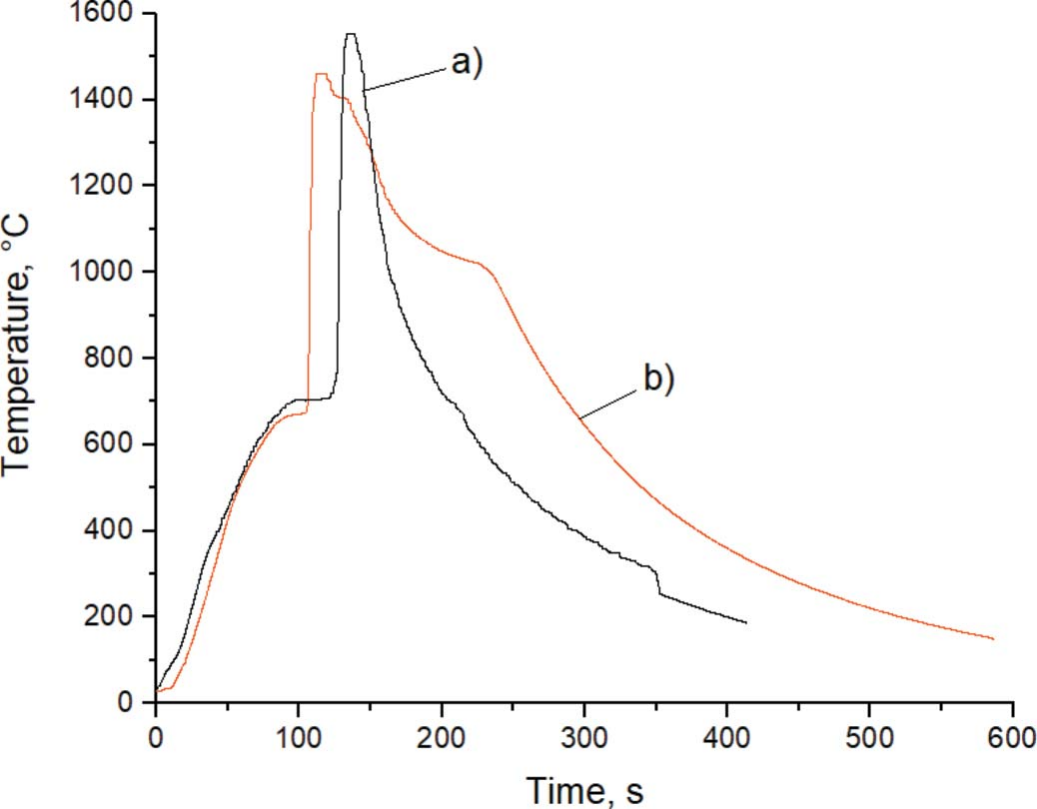
Thermograms of high-temperature synthesis of Ti + Al precursors (MA, *t* = 7 min) followed by their cladding (deposition time τ = 40 min): (*a*) with shutdown; (*b*) with system annealing.

At the first stage, model experiments on the high-temperature synthesis of the Ti + Al clad mechano­composites were performed. The synthesis was carried out in the thermal explosion mode, switching off the heating source when the maximum temperature was reached, and with system annealing after the chemical reaction was over. The continuous transition from the process of high-temperature synthesis to high-temperature annealing at temperatures above adiabatic combustion promotes the transition of the synthesis product to an equilibrium state, which is accompanied by structural relaxation processes and the possibility of obtaining a monophase compound (Filimonov *et al.*, 2014*b* ▸). Fig. 4 ▸ shows characteristic thermograms of high-temperature synthesis.

Based on the results of the series of experiments, the average mixture heating rate was calculated (about 525 K min^−1^). The ignition temperatures corresponded to *T*_ig_ = 650 ± 10°C, and the maximum synthesis temperatures *T*_max_ = 1380–1465°C.

For a detailed study of *in situ* phase formation in the synthesis of clad precursors of Ti 64 wt% + Al composition, synchrotron radiation time-resolved diffractometry was applied. It should be noted that similar studies were previously conducted for mechano­activated samples of the same composition, but without the deposited shell on them; the results are given by Loginova *et al.* (2019*a* ▸).

**Figure 5:**
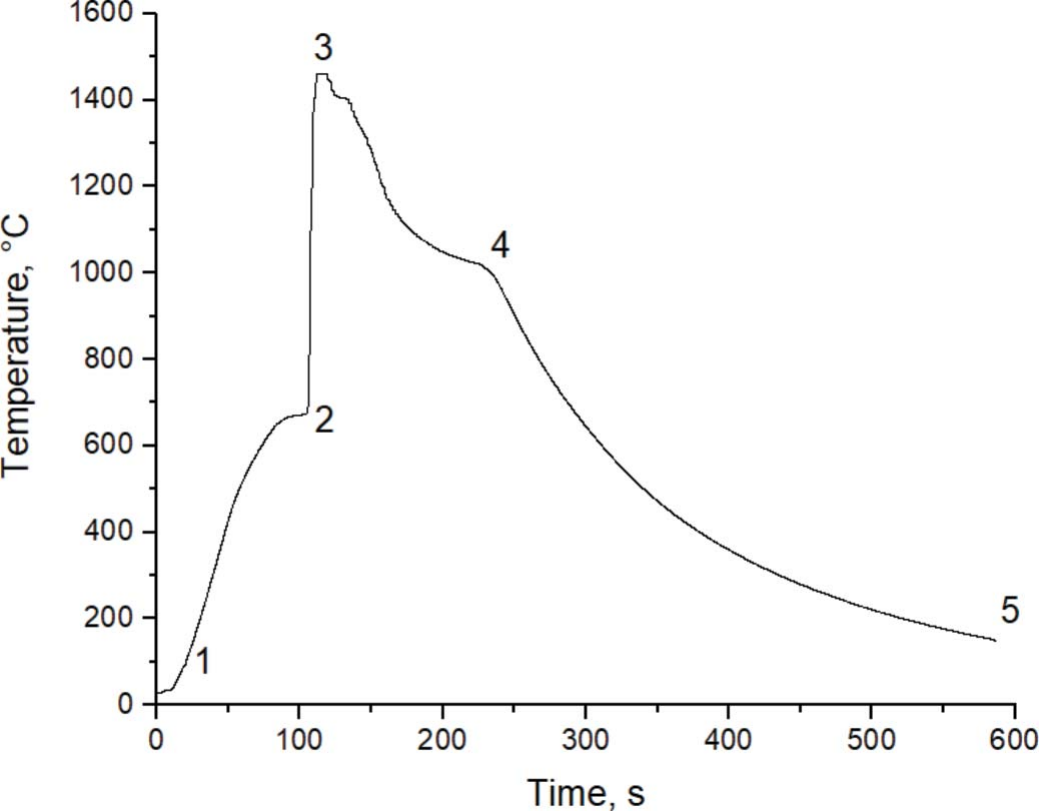
Thermogram of the *in situ* process of high-temperature synthesis of Ti (64 wt%) + Al powder mixture (MA, *t* = 7 min, 40*g*, cladding τ = 40 min).

For convenient analysis, the thermogram presented in Fig. 5 ▸ was divided into characteristic sections: powder mixture heating (1–2), thermal explosion conduction (2–3), annealing (3–4), and cooling (4–5). Next, *in situ* phase-formation analysis was carried out for each section of the synthesis process. The synthesis was initiated starting from temperature *T* = 661°C, corresponding to the melting temperature of aluminium. The maximum reaction temperature is *T*_max_ = 1465°C. In order to study the phase composition after the chemical reaction is over, the heating element was not switched off after reaching the maximum temperature. The annealing time corresponded to *t* = 52 s.

Next, let us consider in more detail the processes of phase formation at the synthesis sector.

### 3.1. Sector 1–2: heating of the powder mixture

Twenty diffractograms were recorded *in situ* in the heating sector of the powder mixture (1–2) up to the characteristic breaking point 2 in the thermogram, characterizing the beginning of the thermal explosion. They were recorded at *T* = 661°C (the time from the beginning of mixture heating *t* = 91.7 s).

**Figure 6:**
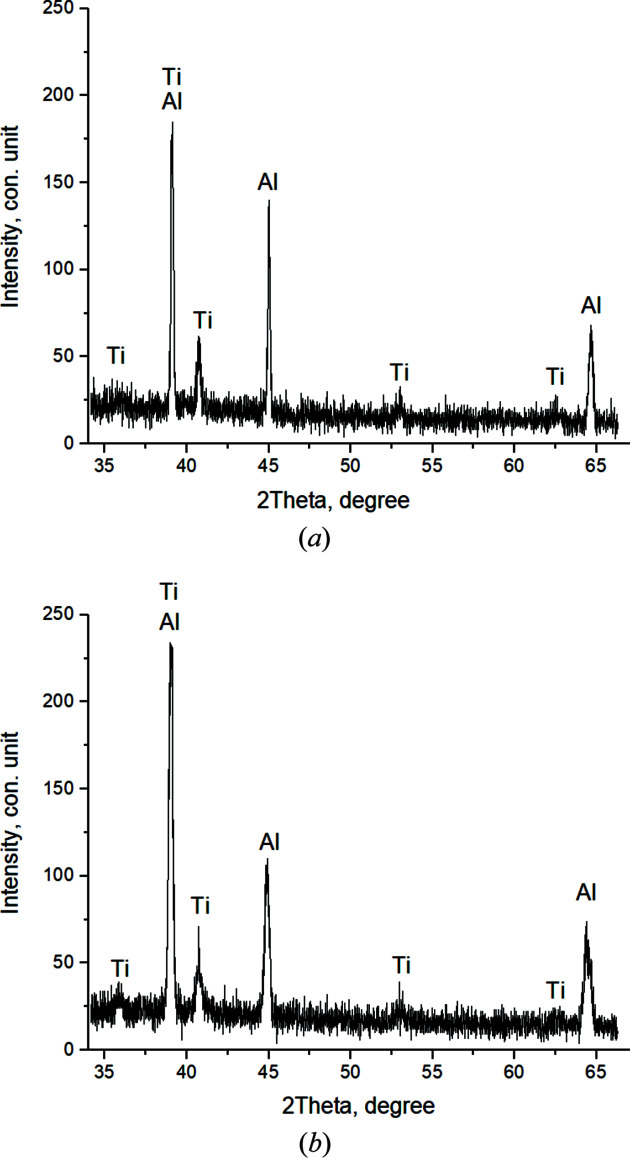
Diffractograms of the 1st (*T* = 410.7°C) (*a*) and the 15th (*T* = 615.7°C) (*b*) shot.

In the first frame [Fig. 6 ▸(*a*)] (*T* = 410.7°C, *t* = 51.7 s), the reflections of the Ti and Al initial components are recorded. The broadened with low-intensity peaks qualitatively testify to the instability of the structural state of the crystal cells (high level of microstresses, small crystallite sizes). Ti (002) and Al (111) contribute to the maximum peak with *I*_max_ = 185 c.u. intensity (where c.u. means conventional units). Then, the values of the main peak intensity slightly changed from *I*_max_ = 155 to 235 c.u. during *t* = 79.7 s to the 16th shot while the phase composition remained unchanged [Fig. 6 ▸(*b*)]. It should be noted that, as the temperature increases, the atoms in the crystal cell of the components oscillate and the interplanar distances change. This leads to a shift of the diffraction reflections to the region of smaller angles, corresponding to an increase of the periods of the elementary aluminium and titanium cell by 0.02 Å.

**Figure 7:**
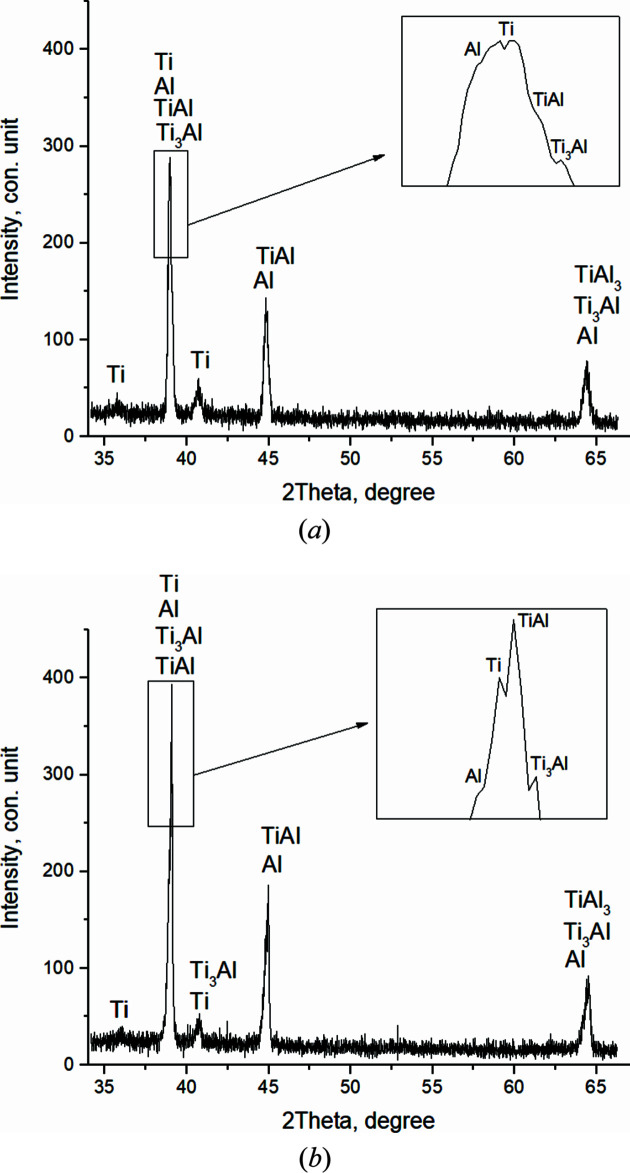
Diffractograms of the 16th (*T* = 620.7°C) (*a*) and the 20th (*T* = 656.5°C) (*b*) shot.

When the heating temperature reaches *T* = 620.7°C (shot 16, *t* = 81.7 s), the intensity values of the main peaks increase sharply (*I*_max_ = 318 c.u.) [Fig. 7 ▸(*a*)] and continue to increase until thermal explosion begins.

In the 20th frame (*T* = 656.5°C, *t* = 89.7 s) [Fig. 7 ▸(*b*)], splitting and asymmetry of diffraction peaks are clearly observed. At the same time, *I*_max_ increases to 392 c.u. This indicates the beginning of compound formation at temperatures close to the melting temperature of the fusible component, *T*_mp_(Al) = 660°C.

For a more detailed representation of the experimental data presented in Fig. 7 ▸, the refined interplanar phase distances included in the main peak are given in comparison with the reference values in Table 1 ▸.

Since the comparison of the experimental and reference values of interplanar distances (*d*) was made taking into account the measurement error, the *d* determination error, over the entire angular range of the shooting, can be considered as given in Table 2 ▸.

**Figure 8:**
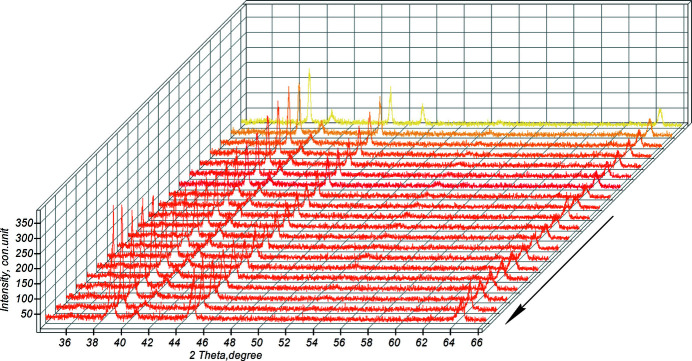
The sector of powder mixture heating during high-temperature synthesis (the arrow indicates the direction of the reaction).

Fig. 8 ▸ shows the diffraction projection, shot in sector (1–2), of the powder mixture heating.

It was found that the formation of TiAl, Ti_3_Al and TiAl_3_ compounds begins after *T* = 656.5°C, practically from the melting temperature of the Al fusible component.

### 3.2. Sector 2–3: thermal explosion

At the stage of thermal explosion (2–3) (Fig. 5 ▸), 12 shots were registered *in situ*. The stage of chemical reaction with the beginning of thermal explosion begins with the characteristic break point corresponding to the melting temperature of Al, *T* = 661°C (*t* = 91.7 s). From the 30th shot from the beginning of the shooting, a sharp temperature increase was recorded (*T* = 775°C, *t* = 109.7 s). The temperature (when the reaction is over) (point 3 in the thermogram of Fig. 5 ▸) corresponds to *T*_max_ = 1465.5°C (*t* = 115.7 s), shot 33; the duration of the reaction is 6 s.

**Figure 9:**
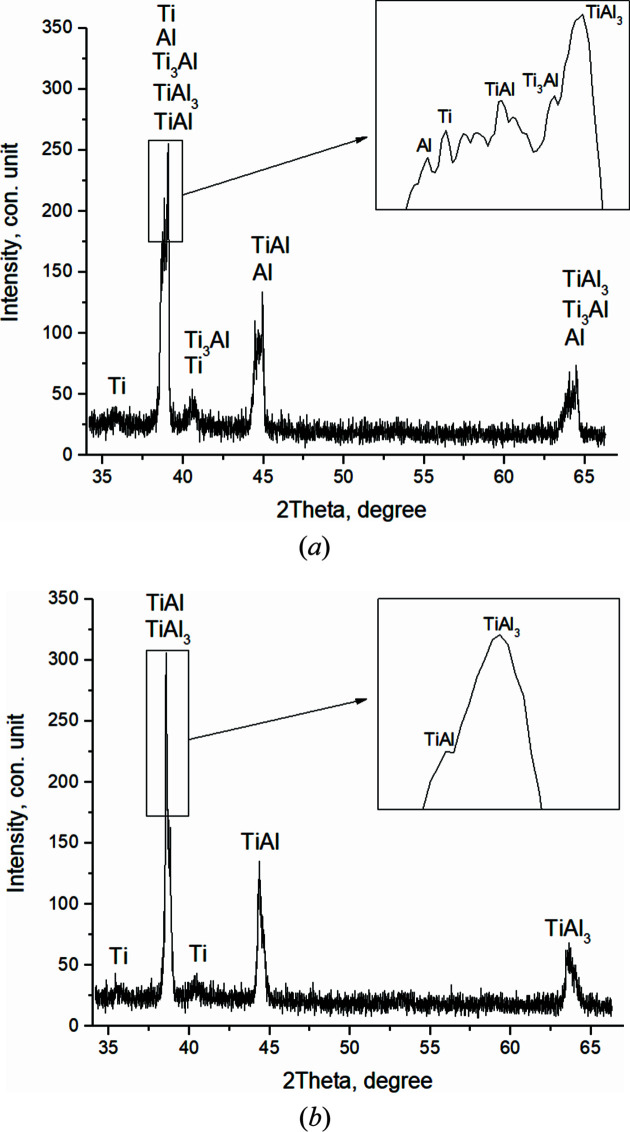
Diffractograms of the 21st (*T* = 661°C) (*a*) and the 22nd (*T* = 662.9°C) (*b*) shots.

Fig. 9 ▸(*a*) shows the diffractogram of the 21st shot, at *T* = 661°C, corresponding to the Al melting temperature. At the same time, there is a decrease in the peak intensities, their splitting, broadening and asymmetry. The intensity of the main peak decreases to *I*_max_ = 255 c.u. Peaks of residual Ti and Al, as well as TiAl, Ti_3_Al and TiAl_3_ compounds, are observed. On the next, the 22nd, shot, *T* = 662.9°C (*t* = 93.7 s) [Fig. 9 ▸(*b*)], there is a slight shift in the angular locations of the peaks. The main peak includes TiAl_3_ (112) and TiAl (111), *I*_max_ = 307 c.u. The diffractogram also shows residual Ti.

**Figure 10:**
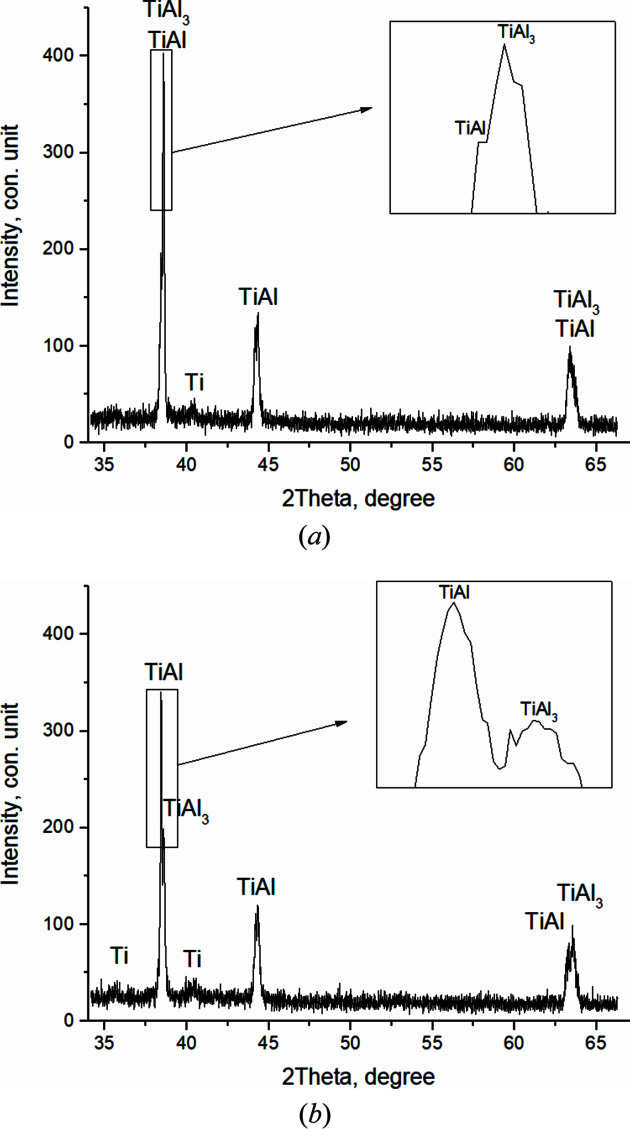
Diffractograms of the 29th (*T* = 674°C) (*a*) and the 31st (*T* = 1184.7°C) (*b*) shots.

A similar picture, with the preservation of the phase composition, is observed during the next 12 s until *T* = 670.9°C, then after (*t* = 107.7 s) there is an increase in the intensity of the main peak up to *I*_max_ = 405 c.u. It includes the reflections of two compounds – TiAl and TiAl_3_ [Fig. 10 ▸(*a*)].

Further, there is a sharp increase in temperature and reaction rate (thermal explosion). At *T* = 1184.7°C, *t* = 111.7 s [Fig. 10 ▸(*b*)], in the peak with the highest intensity the splitting of the main peak is clearly observed, with the corresponding intensities *I*_max_TiAl (111) = 340 c.u. and *I*_max_TiAl_3_ (112) = 200 c.u., which is qualitative evidence of the prevalence of the TiAl content at this stage of the reaction (Fig. 13). There is also a residual Ti.

Tables 3 ▸ and 4 ▸ present the refined interplanar distances of the main peaks in Figs. 9 ▸ and 10 ▸.

Then, when the temperature increases up to *T* = 1406.5°C, *t* = 113.7 s, the intensity values in the main split peak change, *I*_max_TiAl (111) = 155 c.u. and *I*_max_TiAl_3_ (112) = 303 c.u. [Fig. 10 ▸(*a*)]. At this stage TiAl_3_ becomes the dominant phase. TiAl and residual Ti are recorded.

**Figure 11:**
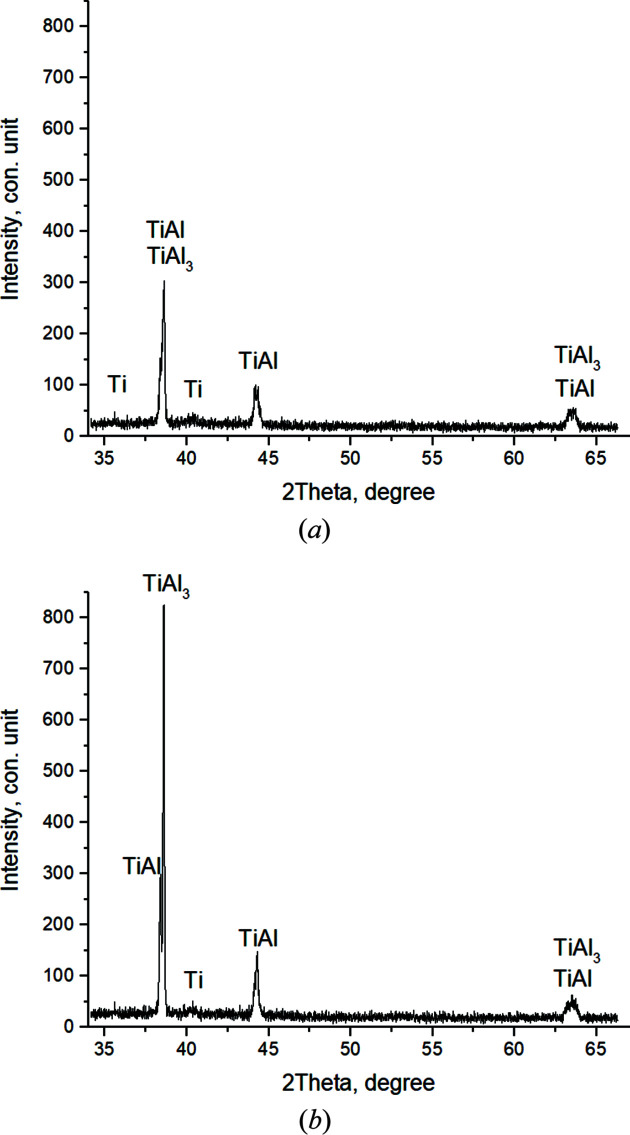
Diffractograms of the 32nd (*T* = 1406.5°C) (*a*) and the 33rd (*T* = 1465.6°C) (*b*) shots.

When the system reaches the maximum temperature *T* = 1465.6°C, *t* = 115.7 s, the reflection intensity of TiAl_3_ (112), which is part of the split main peak *I*_max_TiAl_3_ (112) = 825 c.u., sharply increases. With *I*_max_TiAl (111) = 300 c.u., Ti is also recorded [Fig. 11 ▸(*b*)]. The broadened diffraction maxima in the diffractogram of the reaction product indicate the presence of nonequilibrium defects which failed to relax as a result of the high rate of the chemical reaction, exceeding the relaxation rate of nonequilibrium defects.

**Figure 12:**
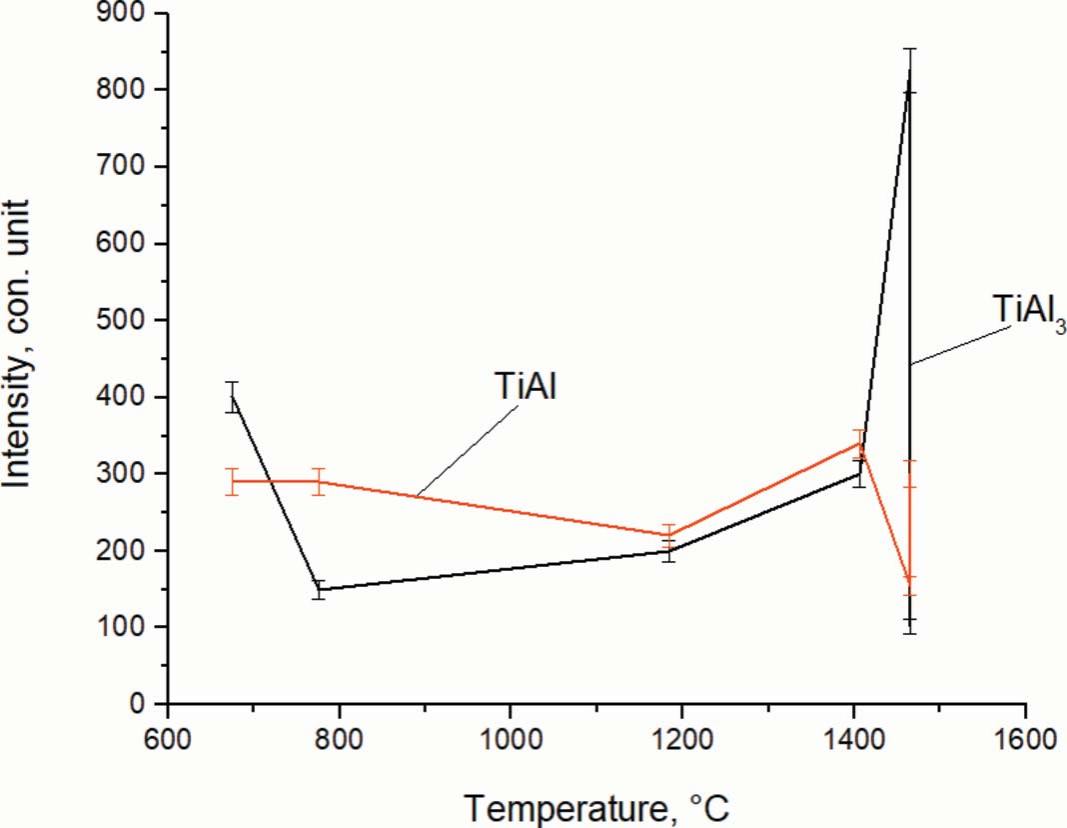
Change of TiAl (111) and TiAl_3_ (112) *in situ* intensity of reflections from temperature during high-temperature synthesis at *T* = 674°C until the system reaches a thermal explosion.

Fig. 12 ▸ shows *in situ* dependences of the change in the diffraction maxima intensity of TiAl_3_ and TiAl main phases when the system enters the thermal explosion. The reflections corresponding to TiAl (111) and TiAl_3_ (112) included in the main peak at its splitting (when the system enters the thermal explosion from *T* = 674°C to *T* = 1465.6°C) were taken as diagnostic reflections.

When determining the intensity errors of the main phases in Figs. 12 ▸ and 16, it was taken into account that during the shooting the signal intensity (number of pulses) in the *i*th channel was *J*, then the absolute error of this value (standard deviation) δ*J* is equal to 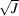 : δ*J* = 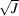 . Taking this into account, the calculation for the background level is δ*Jf* = 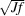 . The noise fluctuation level δ*J* corresponds to 2δ*Jf*. It should be noted that the intensity measurement error is related to the error in determining the position and width of the peaks, since the peak shape is distorted when the intensity decreases. After conducting a comparative analysis of the results obtained with reference values, it was revealed that the discrepancy in the peaks position basically has an accuracy of 0.1° [2θ(exp) − 2θ(ref) < 0.1°]. However, for weaker peak intensities, the observed discrepancy exceeds 0.1°, which is probably due to the static counting error at low peak intensities.

The dependencies given in Fig. 12 ▸ show that with an increase in the heating rate and a thermal explosion of the system there is a sharp increase in the intensity of TiAl_3_ (112), which in 8 s increases up to a maximum value of *I*_max_ = 826 c.u., which qualitatively indicates an increase in the volume fraction of TiAl_3_ intermetallic compound in this section. In this case, the intensity of TiAl (111) has small values, *I*_min_ = 155 c.u.

**Figure 13:**
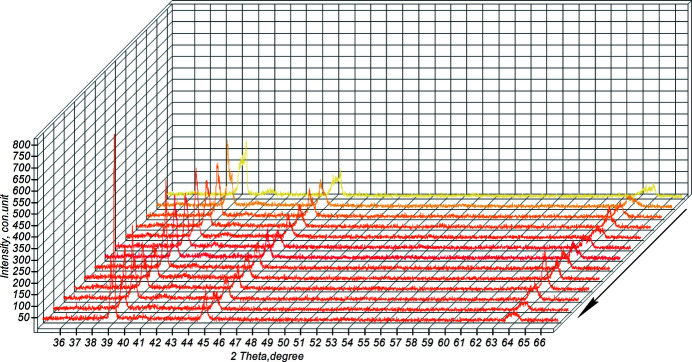
The thermal explosion section (the arrow indicates the direction of the reaction).

Fig. 13 ▸ shows the diffraction projection, taken in the section (2–3) of the thermal explosion. Thus, at a relatively high heating rate of the system, within 6 s from the moment the system explodes and when the system reaches its maximum temperature *T*_max_ = 1465.6°C, the synthesis product is multiphase, with the presence of nonequilibrium defects in the structure. The main phase is TiAl_3_; there is also TiAl and a small amount of Ti.

### 3.3. Section 3–4: system annealing

After the chemical reaction was over, the system was annealed to study the secondary processes of phase formation. At the exposing stage, *t* = 52 s (3–4), *in situ* 26 shots were registered.

During the first 2 s at *T* = 1443.9°C at the exposing stage there is a decrease in the intensity of diffraction maxima and an increase in the level of diffuse background, so *I*_max_TiAl_3_ (112) = 100 c.u. and *I*_max_TiAl (111) = 75 c.u. are recorded in the main split peak. Ti peaks are difficult to identify, since they are at the background level.

**Figure 14:**
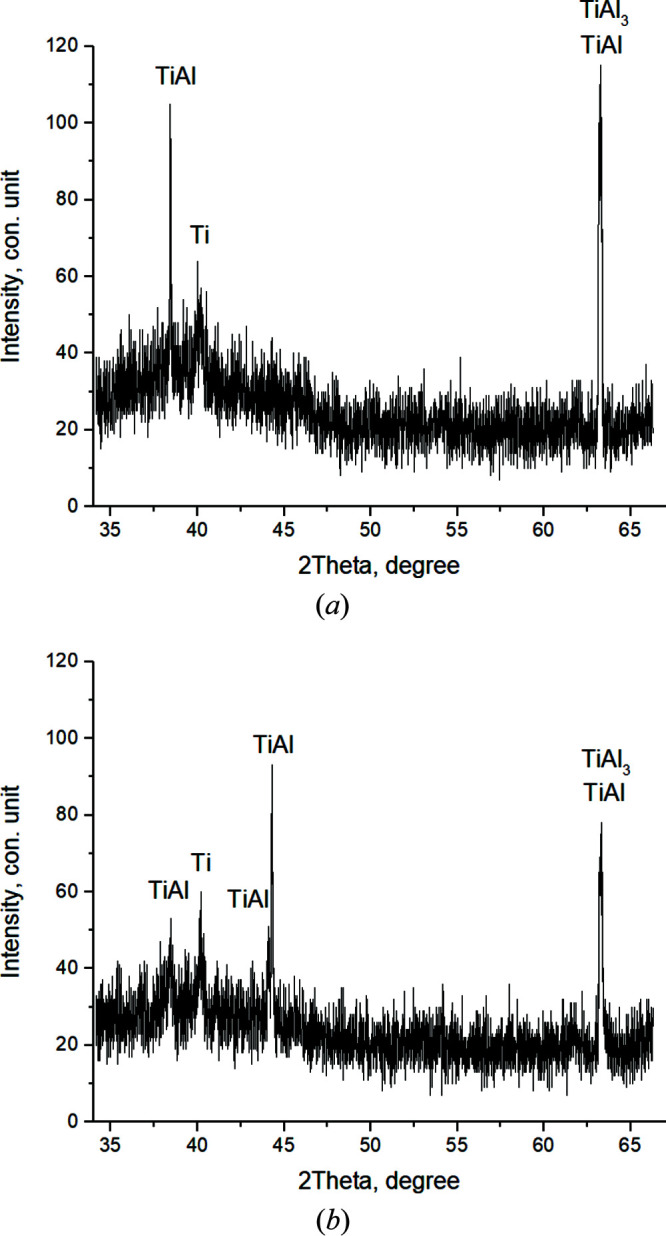
Diffractograms of the 36th (*T* = 1405°C) (*a*) and the 39th (*T* = 1385°C) (*b*) shots

At 6 s from the beginning of annealing at *T* = 1405°C (*t* = 121.7 s), TiAl_3_ disappears from the main peak. TiAl_3_ along the (204) plane is recorded in a secondary peak at high angles. In this case, *I*_max_TiAl (111) = 100 c.u.; Ti is also present [Fig. 14 ▸(*a*)].

**Figure 15:**
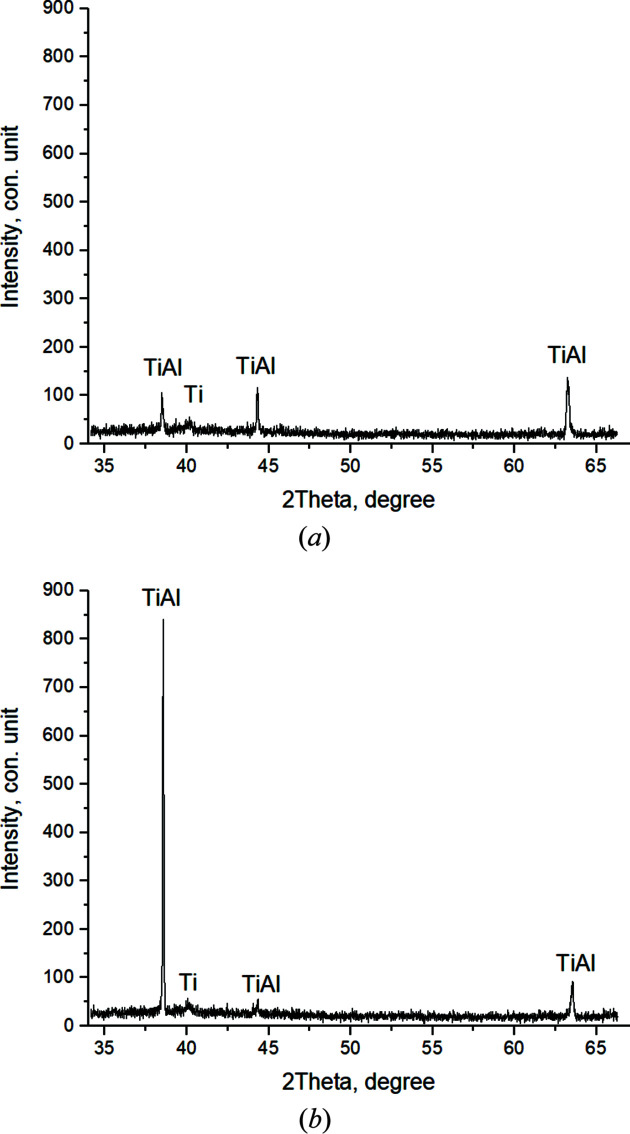
Diffractograms of the 40th (*T* = 1392.9°C) (*a*) and the 41st (*T* = 1384.9°C) (*b*) shots.

After further annealing at *T* = 1394.4°C (*t* = 125.7 s), TiAl content in the product begins to grow, which is qualitatively shown by the appearance of additional TiAl reflections on the planes (002) and (200) and an increase in the intensity of diffraction reflections of this phase [Fig. 14 ▸(*b*)]. TiAl_3_ and Ti are also present. Further, a small temperature jump *T* = 1392.9°C (*t* = 129.7 s) is recorded; on the thermogram (Fig. 5 ▸) an inflection point is observed in this area. The intensities of TiAl peaks continue to grow. TiAl_3_ disappears, and a small amount of Ti remains [Fig. 15 ▸(*a*)].

**Figure 16:**
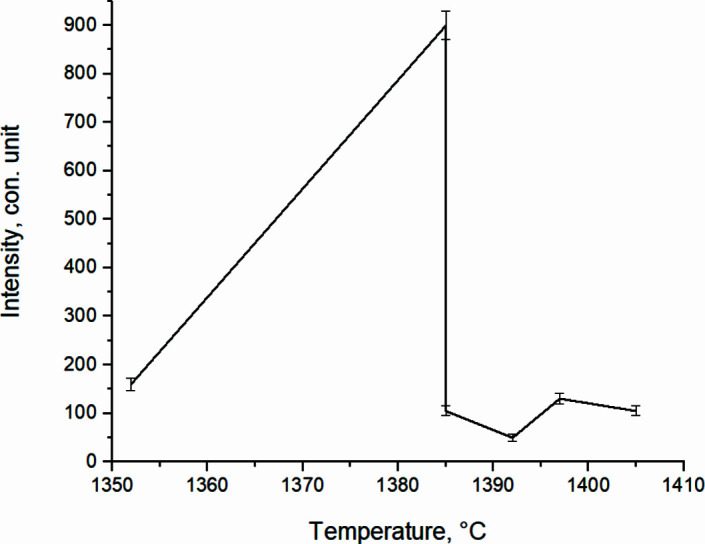
*In situ* variation in the main reflections intensity of TiAl (111) with temperature during the annealing time.

**Figure 17:**
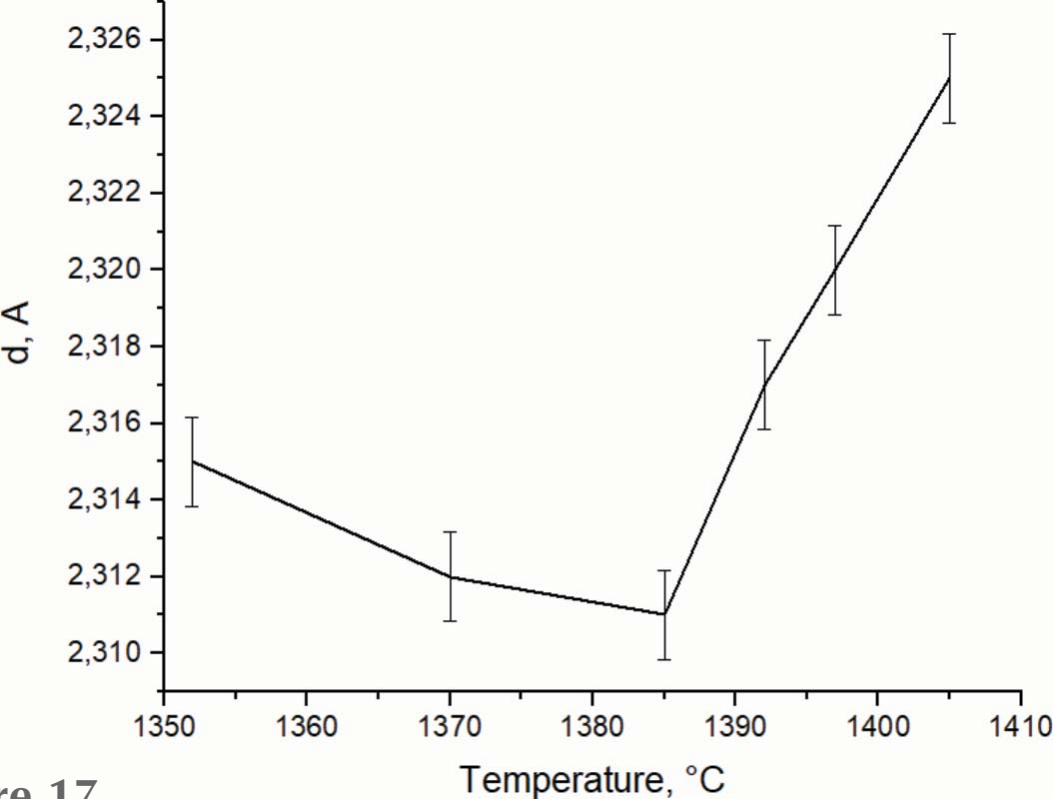
*In situ* variation in the interplanar distances of TiAl (111) with temperature during the annealing time.

In 2 s, at *T* = 1384.9°C (*t* = 131.7 s) [Fig. 15 ▸(*b*)], the diffuse background level decreases, and the intensity of the main TiAl peak (111) increases to the maximum value *I*_max_ = 844 c.u. (Fig. 16 ▸). The half-width of TiAl (111) is 0.026°. At this time the interplanar distance value TiAl (111) (Fig. 17 ▸) is close to the reference *d* = 2.311 Å [reference TiAl (111), *d* = 2.31 Å (card 5–678; Duwez & Taylor, 1952 ▸; PDWin 3.0 International Powder Diffractometry Database)]. The diffractogram also records a Ti (101) reflection of low intensity, *I*_Ti_ = 54 c.u. Therefore, in 16 s of annealing after thermal explosion, an almost monophase TiAl is formed, with a very narrow peak and high intensity, which qualitatively indicates the stabilization of the crystal structure and the formation of stoichiometric TiAl.

The errors in the interplanar distances were determined by differentiating the expression sinθ = λ/(2*d*_HKL_) by θ and by *d*, which gives Δ*d*/*d* = cotθΔθ, where Δ*d*/*d* is the relative error of the interplanar distance; Δθ is the absolute error in measuring the Bragg angle. The error values of the interplanar distances are given in Table 2 ▸.

**Figure 18:**
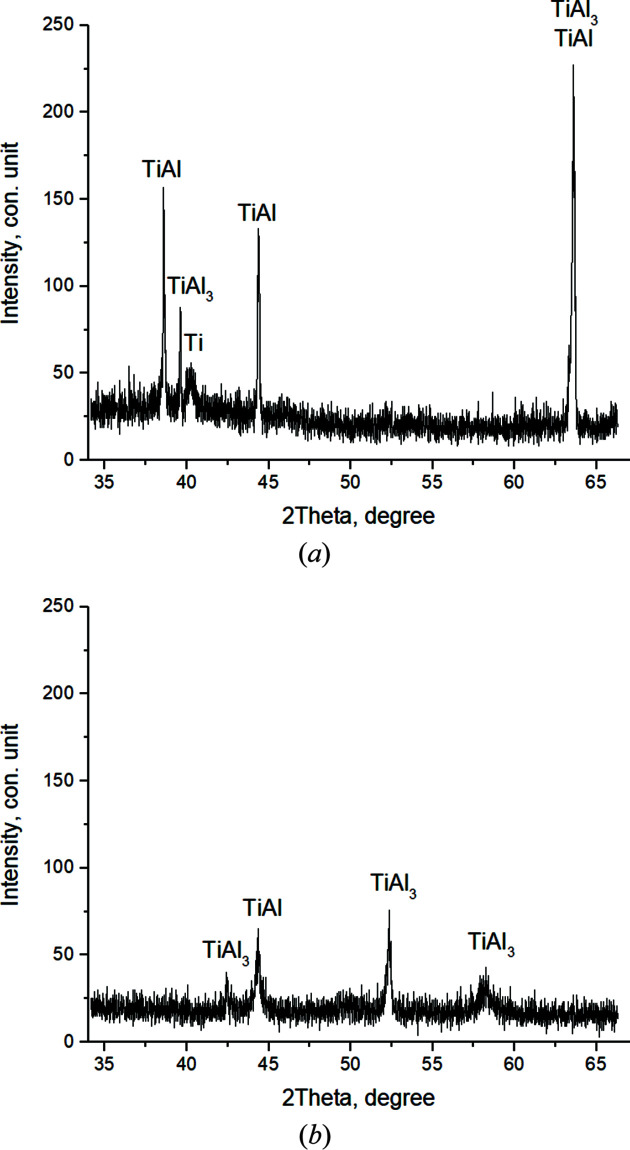
Diffractograms of the 43rd (*T* = 1352.5°C) (*a*) and the 60th (*T* = 1118.6°C) (*b*) shots.

With a subsequent temperature decrease from *T* = 1370.4°C (*t* = 133.7 s), the intensity of the main TiAl peak decreases to *I*_max_ = 581 c.u. An additional TiAl_3_ (204) reflection appears, and the Ti peak is also preserved. Further, the decay of the TiAl phase continues, *I*_max_TiAl (111) = 159 c.u., and diffuse background and the width of the peaks increase. This qualitatively indicates disordered structure; additional TiAl_3_ reflexes appear at the diffractograms [Fig. 18 ▸(*a*)].

Starting from *T* = 1322.3°C (*t* = 139.7 s) until the end of annealing (60th shot, *T* = 1118.6°C, *t* = 169.7 s) [Fig. 18 ▸(*b*)], as a result of further phase transformation, only peaks from two compounds TiAl and TiAl_3_ are recorded in the diffractograms. Moreover, they are in an unstable non-equilibrium state. This is shown by the low intensity of the widened peaks *I*_max_TiAl (200) = 63 c.u. and *I*_max_TiAl_3_ (202) = 77 c.u. and the high level of diffuse scattering.

**Figure 19:**
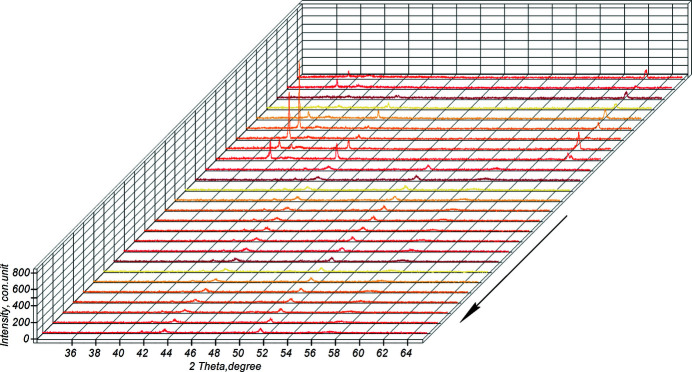
The system annealing section (the arrow indicates the direction of the reaction).

Fig. 19 ▸ shows the diffraction projection taken in section (3–4) of the system annealing. At the stage of system annealing, 16 s after switching off the source, an almost monophase intermetallic TiAl compound of stoichiometric composition is formed. It is qualitatively shown by the TiAl (111) half-width, which is 0.026°, and *I*_max_TiAl = 844 c.u. That is, at the time of the thermal explosion, the phase recrystallization process is not completed, and the TiAl_3_ and TiAl structures have a nonequilibrium state, which is characterized by the incompleteness of diffusion processes. At the stage of secondary structure formation, starting from *T* = 1384.9°C, additional reaction of the components occurs with the formation of almost monophase TiAl. The characteristic inflection point at the thermogram (Fig. 5 ▸), which confirms the processes of secondary structure formation, corresponds to the slow cooling process.

### 3.4. Sector 4–5: system cooling

At the characteristic point corresponding to *T* = 1116.7°C, *t* = 171.7 s (Fig. 5 ▸), the heating source was turned off. At the stage of system cooling (4–5), *in situ* 111 shots were recorded. The last, the 172nd, shot corresponds to *T* = 364.6°C and *t* = 393.7 s.

**Figure 20:**
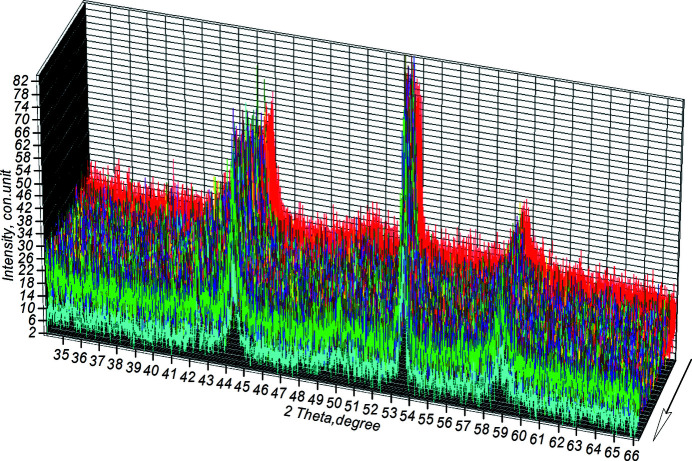
The section of switching off the system (the arrow indicates the direction of the reaction).

Fig. 20 ▸ shows a diffraction projection taken at stage (4–5), when the system was turned off. As follows from the projection, no phase changes are observed after switching off the heating source. *In situ*, two reflections, TiAl and TiAl_3_, are recorded, which remain until the end of the shooting. TiAl_3_ has a shift from plane (202) to (211) in the area of high angles.

The values of the main peaks intensities for TiAl and TiAl_3_ in the cooling area vary within the range from 50 c.u. up to 95 c.u. Low intensity, widened peaks and high diffuse background indicate a non-equilibrium structure of TiAl and TiAl_3_ that persists throughout the entire cooling phase.

**Figure 21:**
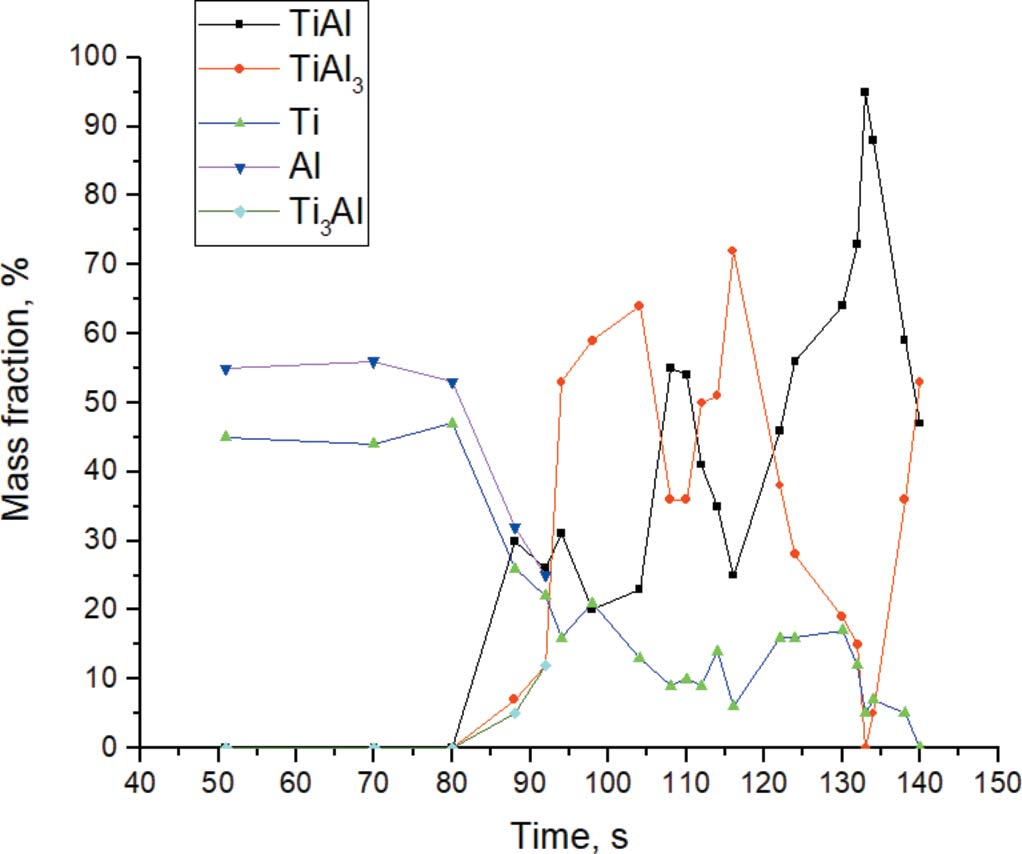
Relative content of *in situ* fixed phases in the process of high-temperature synthesis.

For a qualitative representation of the formation processes of reaction products, Fig. 21 ▸ shows a diagram of the relative content of phases recorded from the beginning of the shooting until the end of thermal explosion realization. It should be noted that it is impossible to make an accurate analysis of the quantitative determination of the content of the reaction products (for example, by the Rietfeld method) due to the conditions of the shooting (relatively low counting speed, limited angular range 2θ = 34–66°) and the noisiness of some diffractograms. However, in order to provide a clearer picture of the changes in the composition of the reaction products during the thermal explosion, a semi-quantitative assessment of the phase composition was carried out, which made it possible to estimate roughly the relative content of the phases by the ratio of the intensities of the maximum peaks, without considering the mass absorption coefficients (Mittemeijer & Scardi, 2004 ▸). The error in this method is 2–3%, taking into account that the statistical counting error increases for small peak intensities; hence, the error bars for weak peak intensities increase. The method of semi-quantitative determination of the relative phase content is based on the intensity dependence of the determined phases *I*_*n*_ diffraction maxima on its content in the *X*_*n*_ sample. The relative phase content can be estimated by taking as a basis a system of (*n* − 1) equations, by solving which the set {*x*_*i*_} was determined:

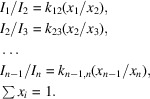

The coefficients *k*_*i*,*i*+1_ were determined by an independent powder mixture shooting with a known phase content (*x*_*i*_/*x*_*i*+1_).

The product formation begins with the Al melting. At 90 s at *T* = 661°C, the relative content of the intermetallic phases is about 25% TiAl, with TiAl_3_ and Ti_3_Al forming about half as much. At 116 s, by the time the system reaches the maximum temperature *T*_max_ = 1465.6°C in the multiphase product, the dominant phase by percentage content is TiAl_3_ (about 70%). At the stage of system annealing at *T* = 1384.9°C, almost monophase TiAl is formed (more than 90%); the rest is occupied by Ti.

## 4. Discussion and conclusions

It is known that the ignition temperature of unclad mechano­composites of Ti + Al is lower than the melting temperature of the fusible component, and the initiation of the thermal explosion can occur in the solid phase (Medda *et al.*, 2003 ▸; Yi *et al.*, 1992 ▸) at a high preheating rate in the induction setup. Mechanoactivation pretreatment has also been found to lower the maximum synthesis temperatures by reducing the reaction activation energy (Adeli *et al.*, 2017 ▸; Xu *et al.*, 2006 ▸). Loginova *et al.* (2019*a* ▸) considered in detail the phase transformations for mechano­activated Ti+Al mixture. It was found that the characteristic heating rate is 500 K min^−1^, and the ignition temperature is lower than the melting of the fusible component (about *T* = 555°C). The synthesis is initiated in the solid phase. This is facilitated by the fact that the product formation occurs at the initial stage of the system heating, before the thermal explosion. Both stable TiAl, TiAl_3_, TiAl_2_ compounds and metastable phases are formed. At the stage of thermal explosion there is a rapid increase in the content of the TiAl compound, which forms the basis of the reaction product when the system reaches the maximum temperature of *T*_max_ = 1280°C. The synthesized product also contains TiAl_3_ and residual Ti. At the initial stage of the system annealing, there are no special changes in the product. At the end of the reaction, when the system comes to thermodynamic equilibrium, the product contains TiAl and TiAl_3_.

In the present study, by synchrotron radiation time-resolved diffractometry, the authors found that during the synthesis of Ti + Al mechano­composites, with a SiO_2_ shell deposited on them, reaction temperatures and phase formation processes are different than for mechano­composites without cladding. The reaction start temperature corresponds to the aluminium melting point *T*_mp_ = 660°C, and the maximum synthesis temperatures are higher compared with the samples without cladding (about *T*_max_ = 1380–1465°C). In contrast to mechanically activated Ti + Al mixtures, in which the processes of chemical transformations occur already at the stage of mixture heating, the presence of the shell on the mechano­composites causes diffusion inhibition of the reagents, and the formation of basic TiAl, TiAl_3_, Ti_3_Al compounds begins only with the melting temperature of the fusible component (about *T* = 660°C), that is in the liquid phase. Because of this there is an increase in the heating rate up to 525 K min^−1^ and the growth of the synthesis maximum temperatures. Probably, the rapid heating rate contributes to the fact that when the system reaches the maximum temperature the main phase in the synthesis product is the TiAl_3_ compound, as energetically advantageous. The product also contains TiAl. It should be noted that when studying the processes in the combustion mode (SHS), or under the slow heating conditions, many authors have established (Wang & Zhang, 2006 ▸; Vaucher *et al.*, 2011 ▸) that in the Ti–Al system it is the titanium trialuminide that is the first forming phase at the contact boundary of the reagents. The TiAl_3_ and TiAl compounds formed as a result of the reaction have a non­equilibrium state, which may indicate the incompleteness of diffusion processes. At the next stage (at system annealing), the diffusion processes continue, leading to the recrystallization of the TiAl_3_ compound into TiAl and the formation of the product based on TiAl. Further annealing determines the transition to the thermodynamically equilibrium state of the system. As is the case with the unclad Ti + Al mechano­composites synthesis, the final product contains TiAl and TiAl_3_, since slow heating causes the recrystallization of phases in accordance with the equilibrium diagram of the system (Paransky *et al.*, 1996 ▸; Rawers & Wrzesinski, 1992 ▸).

Based on the results of the experiment, it can be said that the presence of a shell on mechano­composites affects the thermal parameters and the processes of phase transitions during high-temperature synthesis. The results of *in situ* experimental studies are summarized in the following conclusions.

(1) When carrying out high-temperature synthesis in clad mechanical composites of Ti + Al composition, the ignition temperatures corresponded to *T*_ig_ = 650 ± 10°C, the maximum synthesis temperatures to *T*_max_ = 1380–1465°C. The characteristic heating rate was 525 K min^−1^.

(2) From analysis of the diffraction projections it follows that for clad mechano­composites the formation of TiAl, TiAl_3_ and Ti_3_Al compounds begins with the melting point of the low-melting component at about *T* = 661°C. The start of intermetallic compounds formation in the liquid phase mode, unlike mechano­composites without cladding, where the start of the reaction occurs in the solid phase, can be influenced by a deposited film of SiO_2_, which can act as a barrier to the start of the reaction.

(3) At the time of the thermal explosion at *T*_max_ = 1465.6°C, the synthesis product is multiphase; TiAl_3_ (about 70%) and TiAl (about 25%) structures have a nonequilibrium state, which is characterized by incomplete diffusion processes.

(4) At the stage of system annealing at *T* = 1384.9°C, the components are pre-reacted with the formation of practically monophase TiAl (about 90%); the rest is occupied by Ti.

(5) Further temperature reduction characterizes the end of structural changes and system transition to a thermodynamically equilibrium state, which determines further decay of the formed TiAl phase. By the end of the annealing, TiAl and TiAl_3_ with a high degree of structural disorder are recorded.

In this work, the authors conducted detailed experimental studies on the effect of clad mechano­composites of the Ti–Al system on the phase formation processes and thermal characteristics of high-temperature synthesis.

The method of high-temperature synthesis in mechanically activated powder mixtures makes it possible to carry out synthesis in the solid-state combustion mode and obtain nanostructured products under nonequilibrium synthesis processes. The use of clad mechano­composites changes the nature of the reactions. In this regard, for a deeper understanding of the behavior of such systems at the stages of synthesis, and in particular for understanding micro- and macro-kinetic processes, it is necessary to correlate experimental data with model combustion mechanisms in nano­structured heterogeneous systems. In this case, methods related to the mechanisms of self-organization of the structure can provide new insights into the mechanisms of synthesis in the mechanically activated and clad nanostructured systems (Sekhar, 2021 ▸). In particular, dissipative vibrational chemical reactions, *i.e.* the Belousov–Zhabotinsky reaction (Li & Sekhar, 2009 ▸; Sekhar *et al.*, 2010 ▸; Epstein *et al.*, 1999 ▸; Nadagouda *et al.*, 2012 ▸), can be used to study the synthesis mechanisms of such systems. Thus, Sekhar *et al.* (2010 ▸) revealed the relationship between dissociative reactions and the formation of intermetallides in the nanoscale state in synthesized equimolar Ni–Al alloys. It is discussed that it is the dissipative oscillations that create and simultaneously disperse nanoparticles depending on the initial temperature, composition and other process conditions. In this regard, the application of the Belousov–Zhabotinsky reaction in clad mechano­composites of the Ti–Al system may provide new insights into the analysis of synthesis mechanisms and become a separate task.

## Figures and Tables

**Table 1:** Interplanar phase distances for diffractograms in Fig. 7 ▸

Phase (*hkl*)	*d* (Å) (exp) at 620.7°C [for Fig. 7 ▸(*a*)]	*d* (Å) (exp) at 656.5°C [for Fig. 7 ▸(*b*)]	*d* (Å) (reference)
Ti (002)	2.346	2.344	2.344
Al (111)	2.340	2.339	2.338
TiAl (111)	2.316	2.313	2.312
Ti_3_Al (002)	2.310	2.309	2.307

**Table 2:** Error in determining interplanar distances

*d* (Å)	3.0–2.0	2–1.5	1.5–1
Δ*d* (Å)	0.02–0.007	0.007–0.003	0.003–0.001

**Table 3:** Interplanar phase distances for diffractograms in Fig. 9 ▸

Phase (*hkl*)	*d* (Å) (exp) at 661°C [for Fig. 9 ▸(*a*)]	*d* (Å) (exp) at 662.9°C [for Fig. 9 ▸(*b*)]	*d* (Å) (reference)
Ti (002)	2.347	2.346	2.344
Al (111)	2.340	–	2.338
TiAl (111)	2.316	2.315	2.312
Ti_3_Al (002)	2.310	–	2.307
TiAl_3_ (112)	2.300	2.302	2.301

**Table 4:** Interplanar phase distances for diffractograms in Fig. 10 ▸

Phase (*hkl*)	*d* (Å) (exp) at 674°C [for Fig. 10 ▸(*a*)]	*d* (Å) (exp) at 1184.7°C [for Fig. 10 ▸(*b*)]	*d* (Å) (reference)
TiAl (111)	2.314	2.313	2.312
TiAl_3_ (112)	2.290	2.300	2.301
